# Obstructive sleep apnoea syndrome and anatomical factors: possible correlations

**DOI:** 10.5935/1984-0063.20220074

**Published:** 2022

**Authors:** Giovanna Maino, Francesca Cremonini, Giulia Pettinato, Emanuele Paoletto, Luca Lombardo

**Affiliations:** University of Ferrara, Postgraduate School of Orthodontics - Ferrara - Italy

**Keywords:** Sleep Apnea Syndromes, Sleep, Orthodontics

## Abstract

**Objectives:**

The following retrospective study was devised with the aim of evaluating the correlation between OSAS and various anatomical factors.

**Material and Methods:**

Thirty-seven patients over the age of 40 were analyzed, of which 19 were classified as OSAS cases and 18 as control cases. For each, 17 anatomical variables were identified and examined using Invivo Dental software on CBCT scans, WebCeph software on laterolateral teleradiographs, and Rhinoceros 6.0 software on dental casts.

**Results:**

A generalized linear model of all the anatomical factors identified only two statistically significant variables. Specifically, the total volume of the palate displayed a inverse correlation with OSAS, while the distance between the S point and the Go point (S-Go) exhibited a direct correlation with the disease.

**Conclusion:**

The likelihood of an individual having OSAS appears to decrease as the volume of the palate increases but increase as the lingual measure S-Go increases.

## INTRODUCTION

Obstructive sleep apnoea syndrome (OSAS) is a chronic disorder characterized by partial or total obstruction of the upper airways during sleep. Despite the respiratory effort to overcome the effects of the obstruction, there is an increase in the partial pressure of carbon dioxide and a fall in the partial pressure of oxygen in the blood. To end the apnoeic event and restore the patency of the respiratory tract, a protective mechanism called “arousal”, or micro-awakening, is triggered by the cortical system^1,2^. OSAS is diagnosed by means of the AHI index, i.e., the number of apnoeic/hypopnoeic events per hour during sleep. An AHI of between 5 and 14 denotes mild OSAS, AHI 15 to 29 moderate, and AHI 30 or higher severe^3^. The gold standard diagnostic examination for apnoeas/hypopneas is polysomnography, but cardiovascular monitoring and relevant questionnaires may also be helpful^4^.

The substantial difference between OSAS and OSA (obstructive sleep apnoea) is the presence in the former of a set of daytime symptoms, including morning OSAS, headache, tiredness or fatigue, difficulty concentrating, memory disturbance, daytime sleepiness, and falling asleep during the day^1-2^. Moreover, previous review evidenced that sleep disorders matter in terms of cardiometabolic health and contribute to metabolic syndrome^5^. The severity of OSA is direct correlated to the risk of several disease comorbidities, including hypertension, diabetes, obesity, heart disease, and poor mental health^6^.

Bearing in mind the severity of the condition, several different treatment options are available, to be used alone or in combination. In milder forms, behavioral therapy^7^ or positional therapy^8^ may be sufficient, whereas mandibular advancement devices (MADs) may be required in cases of moderate severity^9^. In most severe forms, the gold standard therapy is ventilatory therapy with CPAP^10,11^, but surgery may be warranted in some cases^6^.

Identification of anatomical and non-anatomical risk factors that contribute to the development of OSAS plays a fundamental role in both treatment selection and prevention of this pathology.

However, the literature contains few studies analyzing the relationship between OSAS and anatomical factors, taking into account a very limited number of variables. For sure, a relationship between craniofacial disharmony and OSAS exists and it is supported by a recent systematic review with meta-analysis.

More specifically, strong evidence for reduced pharyngeal airway space, inferiorly placed hyoid bone and increased anterior facial heights is found in adult OSA patients compared to control subjects^12^.

To further this line of research, a case-controlled retrospective study was conducted to investigate the potential links between OSAS and several anatomical factors measured on laterolateral teleradiography and cone beam computed tomography (CBCT) scans and dental models from patients with and without OSAS.

## MATERIAL AND METHODS

The study design was performed in accordance with the 1975 Declaration of Helsinki ethical standards and its later amendments, and comparable ethical standards. It was approved by the Ethics Committee of the Ferrara University Postgraduate School of Orthodontics (Via Luigi Borsari 46, Ferrara, Italy; approval number 6/2021).

Data from 37 subjects, 19 with OSAS (15 males and 4 females) and 18 controls without (16 females and 2 males) were analyzed. All patients were being treated by the same dentist at the same clinic. Patients included in the study group met the following criteria:

Aged between 40 to 50 years old;

BMI<30kg/m^2^;

Diagnosis of moderate or severe OSAS (AHI>15), as assessed by polysomnography or cardiorespiratory monitoring;

Patients included in the control group met the following criteria:

Aged between 40 to 50 years old;

BMI<30kg/m^2^;

Absence of symptoms indicative of OSAS;

Assigned to the “low risk” group by responses to the Berlin questionnaire.

**Figure 1:**
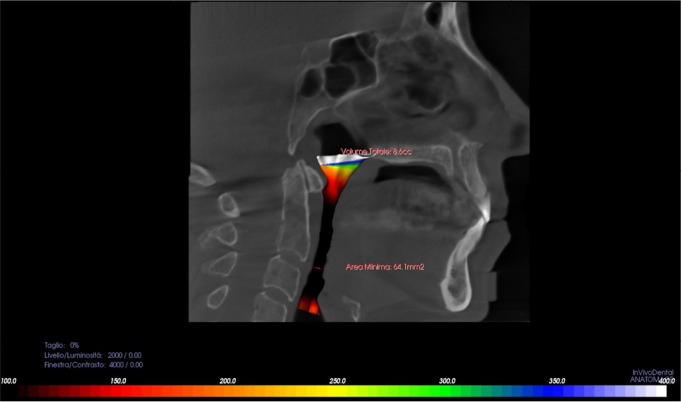
Representation of measurements of total airway volume and minimum airway cross-section using InVivo Dental software.

The control group initially consisted of 20 subjects, two of whom were excluded from the study because they fell into the “high OSAS risk” category according to the Berlin questionnaire. The variables analyzed for each subject are presented in Table 1. Both groups were given a CBCT scan using the same machine at the same clinic. Subjects were positioned vertically, with the Frankfurt plane parallel to the floor. The resulting images were viewed and analysed using InVivo Dental Software (version 5.2, Anatomage). Specifically, the “Volume Rendering Tab” mode was used to measure the total airway volume in cm^3^ and minimum airway cross-section (narrowest CSA) in mm^2^. As per Momany et al. (2016)^13^, the field of interest was defined on the best sagittal view of the airways, automatically provided by the software, which included a clear view of the posterior nasal spine (PNS) and the second cervical vertebra (C2). The software generates a rendering of the airway volumes using a colour code, and indicates the smallest cross-section of the airways (narrowest CSA) with a red circle (Figure 1). Subsequently, teleradiographs of all patients, already present in the clinic’s digital archives, were subjected to cephalometric analysis by the same operator (G.P.) using the WebCeph digital orthodontic and orthognathic analysis platform.

**Table 1:** Anatomical parameters collected and means of acquisition.

Total volume of the airways	CBCT
Minimal cross section of the airways	CBCT
Skeletal sagittal Class (ANB angle)	Laterolateral teleradiograph
Facial divergence (FMA angle)	Laterolateral teleradiograph
Posterior facial height (S-Go)	Laterolateral teleradiograph
Mandibular length (Go-Me)	Laterolateral teleradiograph
Point H - mandibular plane distance (H-MP)	Laterolateral teleradiograph
Toungue length (T1-TT)	Laterolateral teleradiograph
Upper airways lenght (T1-PNS)	Laterolateral teleradiograph
Maxillary intercanine distance	Digital dental casts
Mandibular intercanine distance	Digital dental casts
Maxillary intermolar distance	Digital dental casts
Mandibular intermolar distance	Digital dental casts
Total volume of the palate	Digital dental casts
Canine dental Class	Digital dental casts
Molar dental Class	Digital dental casts

**Figure 2:**
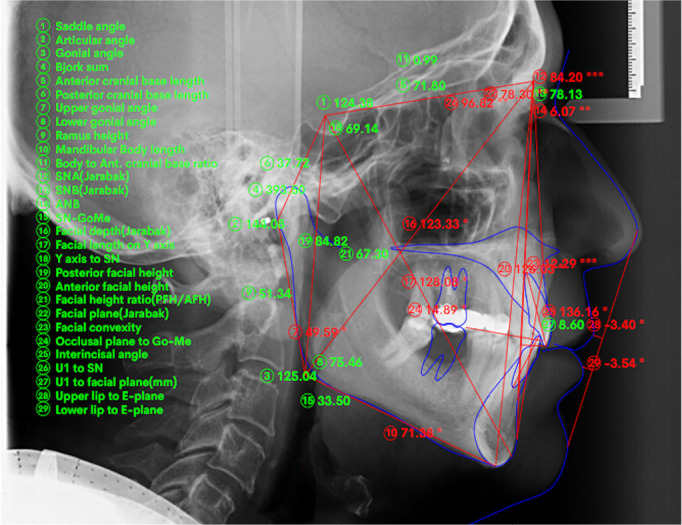
Representation of cephalometric measurements obtained via Webceph.

Plaster models of the dental arches of each study and control group subjects, also stored in the clinic’s archives, were scanned using the Carestream Dental 3600 intraoral digital scanning system. The files obtained from the scan in STL format were then viewed and processed using Rhinoceros 6.0 by the same operator (G.P) (Figure 2). As indicated by the study by Kecik (2017)^14^, the intercanine distance was measured from the highest point of the right canine cusp to the highest point of the left canine cusp, while the intermolar distance was measured from the apex of the mesiovestibular cusp of the first molars to the corresponding contralateral. The molar and canine dental Class was evaluated on digital dental casts and the Angle’s classification of malocclusion taken into consideration.

In patients with missing teeth or prostheses, measurements were taken from the apex of the edentulous ridges or the center of the abutments, respectively.

**Figure 3:**
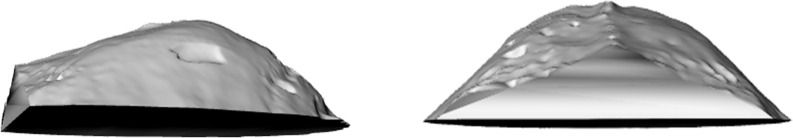
Representation of the discrete solid used to measure the palatal volume in right and posterior views, respectively.

The volume of the palate was measured by tracing two planes on each: first the gingival, by connecting the line connecting the most apical point of the dentogingival junction at all the erupted teeth, and then the distal, perpendicular to the gingival plane and passing through the two most distal points on the distal surfaces of the permanent second molars^14^. Cropping the image at the distal plane and the gingival plane provided a 3D image of the palate, which was transformed into a solid by the software in order to calculate the volume (Figure 3).

### Statistical analysis

A preliminary analysis of the effect of each variable concomitant to the presence or absence of the disease was conducted by examining the empirical distribution of the response to variations in the individual explanatory variables. The graph used for this analysis was the box plot, since the response variable is dichotomous while the explanatory variables are continuous in nature. As the variables “canine Class” and “molar Class” are discrete categorical variables, a histogram was used to study their effect on the response variable. Being qualitative, the modalities of the variables “canine Class” and “molar Class” were codified by assigning them a number from 1 to 16. Subsequently, a generalized linear model appropriate for the present case was estimated.

## RESULTS

**Figure 4:**
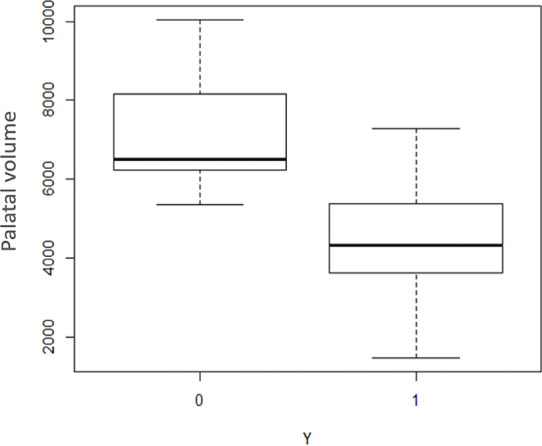
Box plot illustrating the distribution of the “palatal volume” variable for the control group (0) and the study group (1).

**Figure 5:**
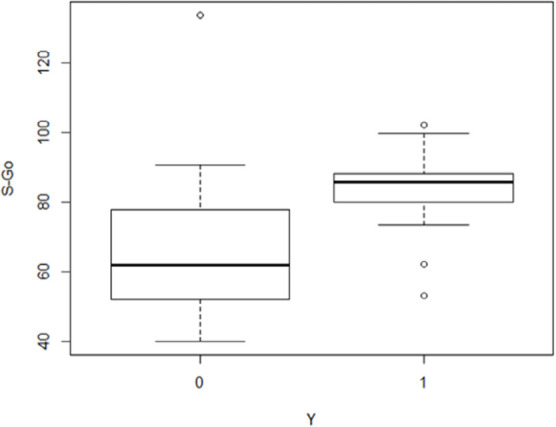
Box plot illustrating the distribution of the “S-Go” variable for the control group (0) and the study group (1).

**Figure 6:**
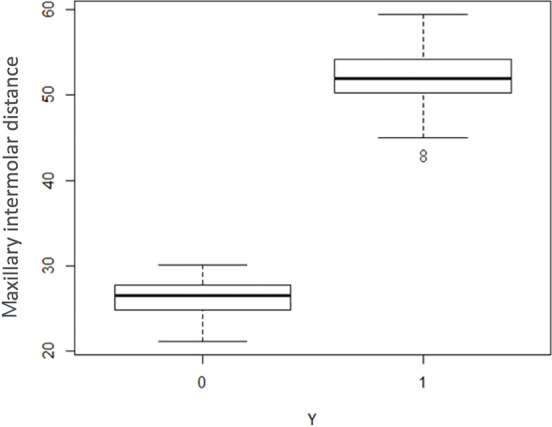
Box plot illustrating the distribution of the variable "Maxillary intermolar distance" for the control group (0) and the study group (1).

**Figure 7:**
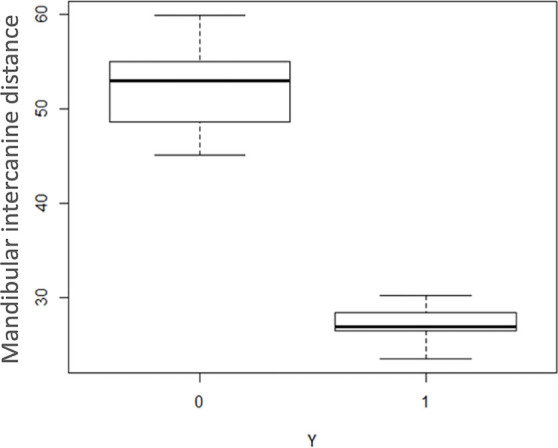
Box plot illustrating the distribution of the variable "mandibular intercanine distance" for the control group (0) and the study group (1).

The preliminary analysis carried out showed a possible significant effect of the variables “total volume of the palate”, “S-Go”, “maxillary intermolar distance” and “mandibular intercanine distance” (Figures 4-7), whereas the other variables studied were not significant. However, R-study adaptation of the specified generalized linear model revealed that the only two variables to be significant at a significance threshold of 5% were “total volume of the palate” and “S-Go”.

The correlation with the variable “total volume of the palate” was inverse; this means that the probability that a subject presents the disease decreases as the volume of the palate increases. The opposite was true for the variable “S-Go”: the probability that a subject presents the disease appears to decrease as the S-Go distance decreases.

The estimated model confirms that deduced from the graphical analysis for the variables “S-Go” and “volume of the palate”. Unlike that indicated by the initial graph, however, the variables “maxillary intermolar distance” and “mandibular intercanine distance” were not found to be significant, probably due to the too small number of subjects examined.

**Figure 8:**
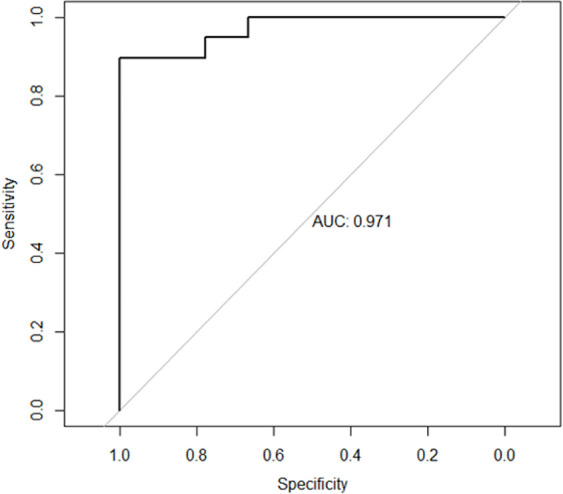
Representation of the ROC curve of the generalized linear model used.

Analysis of the predictive capacity of the estimated model through the ROC curve indicates a good predictive effectiveness, with an area under the curve (AUC) equal to 0.971 (Figure 8).

## DISCUSSION

The aetiology of OSAS encompasses a complex interaction of anatomical and non-anatomical factors. The former lead to a narrowing of the upper airways and represent the main cause of increased risk of airway collapse during sleep. Non-anatomical risk factors are of lesser importance, occurring in only 56% of cases of OSAS and have greater significance in cases of medium severity^15^.

The total airway volume and minimum cross-section of the airways (narrowest CSA) were first analyzed, via CBCT like several other studies in the literature^16-20^. For example, Ogawa et al. (2007)^21^ demonstrated that OSAS subjects have a statistically significant reduction in the minimum cross-section of the airways, positioned below the occlusal plane in 70% of cases, but found no statistically significant difference between the OSAS group and the control group in terms of the total airway volume. Similarly, Bruwier et al. (2016)^22^, by analyzing a larger sample of the population, showed that the minimum cross-section of the airways is lower in OSAS patients than in non-OSAS controls.

A study by Shigeta et al. (2008)^23^ on the other hand, found no statistically significant differences in the minimum cross-section of the airways between OSAS cases and control cases. This is consistent with the results of the current study, albeit contrasting with those cited above. However, it must be emphasized that there were differences in CBCT measurement methods. Specifically, unlike Ogawa et al. (2007)^21^ and Bruwier et al. (2016)^22^ a section of the airways between the PNS and the anteroinferior margin of the C2 vertebra was considered. Indeed, the retrolingual area is less affected by changes in the position of the tongue during imaging, is not affected by changes in size or length of the soft palate, and is therefore a more stable anatomical area with advancing age and increasing BMI^23^.

Another difference was related to the position of the patient during CBCT scan and data acquisition. As a systematic review by Whyte and Gibson (2018)^15^ pointed out, findings from studies using CBCT performed with the patient with OSAS in an upright position should be interpreted with caution. That being said, although the supine CBCT is adequate for OSAS assessment, it provides an incomplete representation of the upper airways. Indeed, the standing position is closer to the natural position of the head (NHP), and is therefore recommended in the guidelines for CBCT evaluation of the morphology and size of the upper airways^24^.

As reported in the literature, laterolateral teleradiography scans, obtained via CBCT, are considered an indispensable method of evaluating the craniofacial and soft tissue features of patients with OSAS, even though it only offers a two-dimensional view of their anatomy^25^. Although some cephalometric parameters that the literature reports as relevant were statistically insignificant in the present study, these should be noted. For instance, a study comparing 10 OSAS patients and 10 control patients found an increased ANB angle and an increased tongue length (T1-TT) in the OSAS group^26^.

Similarly, although a slightly higher FMA has been reported in OSAS^25,26^, according to the above-mentioned data, this is not a parameter significantly related to the disease. In a study by Bacon^27^, on the other hand, it was the length of the maxilla (ANS-PNS) among the cephalometric parameters that was significant; this is likely due to the counter-clockwise rotation of the middle cranial fossa and the palatal plane found in the OSAS subjects, effectively shortening the upper maxilla.

However, a prospective case-control study of patients with OSAS found that only the length of the mandibular body (Go-Me) was significantly reduced, indicating a greater probability of finding a short mandible in OSAS subjects^28^.

There is more consensus on the significance of the distance between the mandibular plane and hyoid bone. Partinen et al. (1988)^29^, found a direct correlation between an increased distance between the hyoid bone and mandibular plane (>18mm) with AHI, and Borges et al. (2013)^30^ and Yucel et al. (2005)^31^ reported similar findings. The latter authors concluded that the position of the hyoid bone has an important impact on the shape and position of the tongue, thereby influencing the flatness of the airways at the level of the hypopharynx. In assessing the correlation of this factor with obstructive sleep apnoea, however, it is necessary to note that the hyoid bone moves forward when shifting from a standing to a supine position^32^.

In the present study, the only cephalometric parameter found to be statistically significant was the posterior facial height (S-Go). This is consistent with findings from other studies, such as those by Ryu et al. (2015)^32^ and Vidovic et al. (2013)^33^. In addition to confirming the above findings, a study by Woodson et al. (1997)^34^ revealed that an increase in posterior facial height is associated with a worse therapeutic response to uvulopalatopharyngoplasty. Indeed, increased length of the pharyngeal airways translates into a greater probability of instability and collapse.

In the present study, some parameters measured on digital scans of dental models were found to be statistically insignificant. Among these were molar and canine dental class, and both maxillary and mandibular intercanine and intermolar distances. Although the majority of OSAS patients examined in the study by Alqahtani et al. (2018)^35^ had molar (43.1%) and canine (49.0%) Angle Class II, there were no statistically significant correlations between occlusion and OSAS severity. Nevertheless, it is interesting that 94.1% of their Class II cases had a division I incisal relationship, especially in light of Banabilh’s contrasting findings of a statistically significant correlation between Angle Class II and OSAS^36,37^.

As regards a potential correlation between OSAS and maxillary and mandibular intercanine and intermolar distances, Seto et al. (2001)^38^ reported that these are shorter in OSAS patients. However, in a study by Johal et al. (2004)^39^ using the same measurement method, no statistically significant correlation was found between interdental distances and OSAS.

In the current study, the only parameter measured on dental models that appears to have a significant correlation with OSAS pathology was the volume of the palate. There are few reports in the literature on measurements of transverse maxillary dimensions combining them with 3D volumetric measurements, but those that do exist indicate that OSAS patients present skeletal modifications of the palatal region that result in a smaller palatal volume than controls^40,41^. Indeed, alterations to the normal physiology of breathing causes a disruption in the balance between the centripetal forces of the cheeks and the centrifugal forces of the tongue. The morphology of the upper jaw is conditioned by tongue posture and function, and it has been shown that changes in physiological nasal and maxillary growth are related to increased resistance at the level of the upper airways^42^. Contraction of the upper jaw also seems to be a compensatory mechanism designed to maintain occlusion in cases in which the jaw is retropositioned, a typical finding in OSAS patients^43^.

Finally, it is important to note that Kecik (2017)^14^ demonstrated the presence of a significant inverse correlation between the area of the soft palate and the palatal volume in OSAS patients, in addition to a direct correlation between nasopharyngeal and oropharyngeal areas and the volume of the palate. This means that upper airway narrowing and lower palatal volume may be specific determinants of OSAS, while a inverse correlation between palatal volume and soft palatal area in OSAS patients is indicative of the altered interaction between hard and soft tissues in these subjects during breathing.

It should also be noted that the data presented here has some limitations. First of all, the reduced sample size represents an important limit of this study, along with the unstandardized selection of patients. Indeed, a consistent difference in gender is present between the study and the control group.

## CONCLUSION

The following statements can be concluded by this retrospective study:

There is an inverse correlation between the OSAS and the volume of the palate, i.e., as the volume of the palate increases, the probability of encountering the pathology in the patient decreases.

There is a direct correlation between the OSAS pathology and the distance S-Go (posterior facial height), i.e., as S-Go increases, the probability of finding the pathology in the patient increases.
